# Clinical and Radiological Outcomes of Paediatric Forearm Fractures of the Radius and Ulna Following Fixation by Intramedullary Nailing or Plating: A Systematic Review

**DOI:** 10.7759/cureus.43557

**Published:** 2023-08-16

**Authors:** Kingsley Mmerem, Mohammad Waseem Beeharry

**Affiliations:** 1 Trauma and Orthopaedics, The Royal London Hospital, London, GBR; 2 Trauma and Orthopaedics, Barts Health NHS Trust, London, GBR

**Keywords:** radius and ulna fractures, clinical outcomes, plating, intramedullary nailing, paediatric fracture, ipsilateral forearm fractures

## Abstract

Ipsilateral forearm fractures of both the radius and ulna in children are one of the most common forms of injuries in this population. They often result from axial loading on the hand and wrist following a fall on an outstretched hand. These injuries can often be managed either conservatively or operatively. Non-operative management involves the use of cast immobilisation after satisfactory closed reduction. Most fractures managed conservatively have been noted to have a successful outcome. Surgical options of management include the use of intramedullary nailing (IMN), plates and screws, hybrid techniques and rarely external fixators.

The purpose of this systematic review is to critically analyse the functional and radiological outcomes as well as the probability of developing a complication in children that have undergone either IMN or plating of both the radius and ulna in the paediatric population. A comprehensive electronic database search from April 2014 until April 2022 was conducted. Studies from PubMed, EMBASE and Cochrane electronic databases were retrieved. A total of 260 cohort studies with children between the ages of 5 to 17 years old were identified. After the application of both inclusion and exclusion criteria, six articles with a total of 409 patients relevant to this review were identified and analysed.

There were no significant inconsistencies statistically in functional and radiological outcomes. Overall complication rate and time to fracture union were similar. Intramedullary nailing was noted to have a shorter operative and anaesthetic time, longer fluoroscopic exposure, and a better cosmetic outcome. Differences in bowing, radial bow magnitude and location had no overall bearing on rotation and daily activity. Considering the methodological limitations of this study, a larger sample size and higher level of evidence such as randomized control studies will yield a more conclusive result to resolve controversies. Based on currently available evidence, both plating and intramedullary nailing are excellent treatment modalities in both-bone forearm fractures.

## Introduction and background

Ipsilateral forearm fractures of both the radius and ulna are fairly common in the paediatric population accounting for under a tenth of all fractures and a third of upper extremity long bone injuries [1-3]. They most commonly result from falling on outstretched hands and records show that twice as many males as females are affected [4,5]. The majority of forearm fractures are traditionally managed conservatively with reduction and cast immobilization as a result of high remodelling potential. Paediatric patients with such fractures, where appropriate moulding technique and three-point fixation was employed, have gone on to achieve an acceptable reduction [3].

However, conservative management in the older paediatric population (10 years of age and older) had less favourable outcomes due to the diminished capacity of inherent remodelling and this is associated with a risk of residual deformity [4]. The functional deficit, in terms of loss of range of motion, arising as a result of these residual deformities remains unclear. Current guidelines suggest a malrotation of 30 degrees or less and an angulation of 10 degrees or less as acceptable [6]. Yet, a few of these forearm fractures would require operative intervention when closed reduction fails or cannot be maintained. Other indications for surgical intervention include open fractures, unstable fractures, pathological fractures, as well as mal-united fractures [1,7]. Surgical management options include the use of intramedullary nailing (IMN), plates and screws, hybrid techniques and rarely external fixators [8].

The concept behind IMN has evolved over the centuries. One of the first IMN systems introduced was the ivory IMN by Heine in 1875. It later got replaced by Gerhard Kuntscher during the Second World War, who sought stability in fixation. The twentieth century also saw the use of other nailing systems which were more flexible. This was later modified to the elastic intramedullary nail, which improved biocompatibility and reduced the risk of mechanical failure [9,10]. The IMN technique of fixation provides a far less invasive approach than plates and screws with minimal soft tissue damage, short anaesthetic and operative time as well as a better cosmetic outcome.

In contrast, plating is considered the gold standard for radius and ulna fractures in the adult population. It offers better anatomical correction of the radial bow, thereby restoring forearm rotation [4]. Over the past few years, there has been an increasing trend towards surgical management which was driven by advancement in biomechanical and medical spheres [6]. However, there are many debates on which fixation method gives a superior outcome. Despite both techniques being suitable for treating both-bone forearm fractures in the paediatric population, there still exist considerable controversies as to which is superior [2]. This review aims to address the difference in functional and radiological outcomes following both operative techniques.

## Review

Methodology

This review was conducted in accordance with the PRISMA (Preferred Reporting Items for Systematic Reviews and Meta-Analyses) guidelines. A comprehensive search of PubMed, Embase and Cochrane electronic databases from April 2014 up to April 2022 was performed. A broad search of literature at different levels was carried out, which compared outcomes of interest in both radius and ulna fractures (IMN versus plating). Search terms employed were “forearm fracture” OR “both bone fracture” OR “radius and ulna” AND “fixation” OR “intramedullary nailing” AND “plating”. A total of 260 studies were retrieved, 12 of which were manually searched by tracing the bibliography of already retrieved articles for additional citations. A critical appraisal checklist was applied to guide the assessment of articles included from the search. A meta-analysis was not deemed suitable due to the small sample size and inconsistency in the pattern of reported outcomes.

Aims

The primary aim is to compare functional and radiological outcomes following IMN and plating in the paediatric population. A secondary aim is to analyse the probability of developing complications associated with either of these techniques.

Eligibility criteria

Inclusion Criteria

The inclusion criteria are as follows: studies published from April 2014 until April 2022, cohort studies, studies published in English, studies comparing IMN versus plate and screws, paediatric population (0 to 18 years inclusive), ipsilateral fractures of both the radius and ulna, closed reduction of greater than 10 degrees angulation.

Exclusion Criteria

The exclusion criteria are as follows: studies considering either IMN or plating and screw, ipsilateral forearm fracture of a single bone, complex fractures (Monteggia, Galeazzi, intra-articular) or pathological fractures, open or mal-united fractures.

Study selection

**Figure 1:**
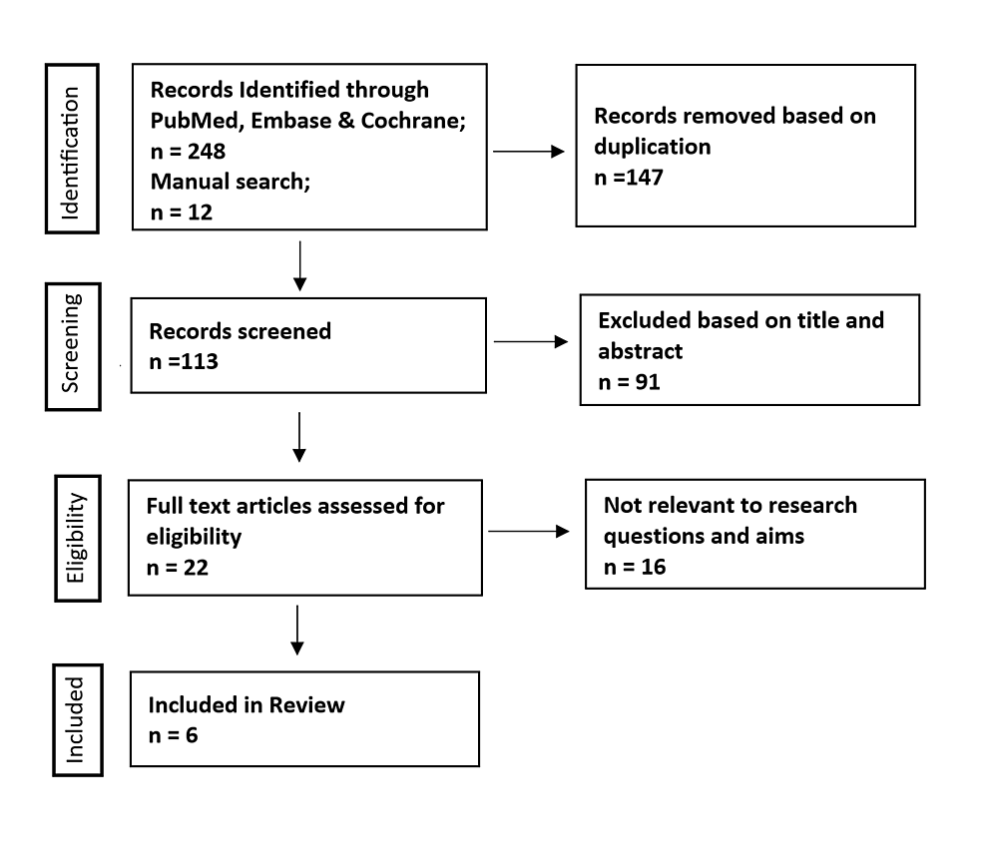
PRISMA flow chart PRISMA: Preferred Reporting Items for Systematic Reviews and Meta-Analyses

A total of 260 studies were identified from the electronic databases Pubmed, Embase and Cochrane as well as through manual searching after application of all inclusion and exclusion criteria. A further 147 studies were removed based on duplication. The remaining 113 studies were screened, of which a further 91 studies were eliminated based on title and abstract. After the elimination of studies due to improper comparison or non-relevance to research questions and aims, only 6 studies were eligible and included in the final review. This was then presented as a flow diagram in compliance with the PRISMA guidelines, illustrated in Figure 1.

Quality assessment

All studies obtained were qualitatively assessed using the Newcastle-Ottawa scale (NOS) [11]. This was qualitatively evaluated for risk of bias in three domains: comparability, selection and outcome of each study. Each study was judged on the eight items as depicted in Table 1. A study could be awarded a maximum of one star for each numbered item within the Selection and Exposure categories. A maximum of two stars could be given for comparability.

**Table 1:** Newcastle-Ottawa scale for assessing the quality of non-randomized trials

Study	Representativeness of the intervention arm	Selection of the comparator arm	Ascertainment of intervention	Demonstration that outcome of interest was not present at the start of the study	Comparability of cohorts on the basis of the design or analysis controlled for confounders	Assessment of outcome	Was follow-up long enough for outcomes to occur	Adequacy of follow-up of cohorts	Score quality
Freese et al. (2018) [4]	*	*	*		**	*	*		7
Thapa et al. (2018) [8]	*	*	*		**	*	*	*	8
Zheng et al. (2018) [1]	*	*	*		**	*	*		7
Zhu et al. (2019) [12]	*	*	*	*	**	*	*		8
Barua et al. (2021) [13]	*	*	*	*	**	*	*		8
Di Giacinto et al. (2021) [14]	*	*	*	*	**	*	*		8

Results

A comprehensive search of the literature identified 260 studies. A total of 147 articles were eliminated as a result of duplicates. A further 91 studies were removed after a review of the title and abstract and subsequently an additional 16 studies on the application of inclusion and exclusion criteria. The full text of one of the relevant articles could not be retrieved from the archives. A total of 6 studies were therefore included in the systematic review as illustrated in the PRISMA flow chart (Figure 1). The studies were largely level III evidence (retrospective and prospective cohort studies). No randomized control trials were involved. The studies were published between 2018 and 2021 and span over a period of 1 to 9 years. The bulk of the study population were adolescents between 10 to 17 years of age who have undergone either IMN or plating following fractures of both the radius and ulna. Patients in both of these groups were followed up for a minimum of 3 months. Outcomes of interest in both groups were assessed using various methods.

A few of the studies showed a reduced time to union with the plating group. However, overall radiological time to union showed no significant difference nor benefit of one group over the other [1,8,12]. Three studies analysed radial bow magnitude and location [4,8,12]. Two out of the three studies that compared plating with IMN had similar bow magnitudes but different locations of the radial bow. The restoration of the post-operative radial bow and location of the maximum radial bow was reported as reduced magnitude and more distal location for IMN [4]. Although not similar to other studies, these parameters are suggested not to affect forearm rotation by some authors [15,16].

Complication rates differed between studies from between 10% to 20.4% in the nailing group and 10% to 20% in the plating group. The overall risk of developing a complication amongst the IMN participants and the plating group were 21.4% and 18.5% respectively, which was not statistically significant. All except one of the studies recorded surgical site infection (SSI) as a re-occurring complication. Participants with SSI responded to oral antibiotics, regular dressing changes and early removal of the metalwork as soon as an infection was detected especially within the nailing group [1,4,8,12].

Tendon adhesion was a complication featured in the study by Thapa et al. in the IMN group [8]. Other considerations are a reduction in the operating time for nailing compared to plating shown in all selected studies, and a slight increase in the duration of hospital stay with nailing participants compared to their counterparts with plating [13]. Two studies recorded the total fluoroscopy time per treatment which was similar. It showed that on average, twice the fluoroscopy time was needed with nailing than with plating [1,12]. Four studies discussed cosmesis post-surgery. Cosmesis was grossly perceived as a self-report of satisfaction without a standardized scoring system. IMN patients were associated with a better cosmetic outcome when compared to plating patients. This cosmetic perception was considered to be quite valuable as they promote a high patient acceptability for nailing.

Functional outcome was assessed in a majority of the included studies using criteria developed by Price et al. [9]. Considering subjective satisfaction and degree of rotation, Price and colleagues graded outcomes as either excellent, good, fair or poor. Excellent meant no complaint on strenuous activities or less than 100 degrees loss of pro-supination. Good, on the other hand, showed mild complaint with strenuous activity and/or 11-30 degrees loss of forearm rotation whereas fair depicted complaints with daily activity and/or 31-90 degrees loss of forearm rotation. Finally, poor was any other outcome or greater than 90 degrees loss of forearm rotation [9]. One of the studies reported functional outcomes in terms of objective quality of life and wrist function by employing different scoring parameters like the Mayo wrist score [14]. Table 2 below outlines the grading of functional outcomes as well as other characteristics of each study.

**Table 2:** Study characteristics summary BI - Bicycle Injuries; M - Male; F - Female; FOOSH - Fall on Outstretched Hands; IMN - Intramedullary Nailing; Lt - Left; Rt - Right; MVA - Motor Vehicle Accident; NR - Not Recorded; RTA - Road Traffic Accident

Study	Duration	Study design	Population		N	Age (SD)	Sex	Side of injury	Injury mechanism	Time to union (weeks)	Functional outcome
Freese et al. (2018) [4]	9 years	Retrospective comparative study	102 (10-16 years)	IMN	70	12.1	44M 26F	Rt:26 Lt:44	Sports: 68, FOOSH: 1, trampoline: 1	9.7	NR
				Plating	32	14.2	22M 10F	Rt:16 Lt:16	Sports: 32	8.2	NR
Thapa et al. (2018) [8]	5 years	Retrospective comparative study	76 (10-17 years)	IMN	46	12.3 ± 1.96	34M 12F	NR	FOOSH: 46	7.86	No significant difference in forearm loss of rotation
				Plating	30	12.7 ± 2.06	24M 6F	NR	NR	7.33	
Zheng et al. (2018) [1]	4 years	Retrospective cohort study	92 (10-16 years)	IMN	48	13.5 ± 1.9	30M 18F	Rt:20 Lt:28	FOOSH: 27, RTA: 15, Others: 6	10.3 ± 2.3	Excellent 62.6%, Good 27.1%, Fair 8.3%, Poor 2.1%
				Plating	44	13.4 ± 1.9	25M 19F	Rt:24 Lt:20	FOOSH: 28, RTA: 10, Others: 6	9.1 ± 2.2	Excellent 56.8%, Good 31.8%, Fair 6.8%
Zhu et al. (2019) [12]	2 years	Prospective study	56 (10-16 years)	IMN	26	13.27 ± 1.64	15M 11F	Rt:14 Lt:12	FOOSH: 19, RTA: 5, Others: 2	10.81 ± 1.47	Excellent 57.7%, Good 34.6%, Fair 7.7%
				Plating	30	13.33 ± 1.54	15M 15F	Rt:15 Lt:15	FOOSH: 22, RTA: 5, Others: 3	10.42 ± 1.50	Excellent 56.7%, Good 30%, Fair 10%, Poor 3.3%
Barua et al. (2021) [13]	1 year	Comparative non-randomized clinical trial	40 (5-15 years)	IMN	20	10.95 ± 2.41	14M 6F	Rt:10 Lt:10	FOOSH: 13, RTA: 7	7.65 ± 1.09	Excellent 85%, Good 15%
				Plating	20	10.4 ± 2.35	14M 6F	Rt:12 Lt:8	FOOSH: 13, RTA: 7	7.45 ± 1.00	Excellent 80%, Good 15%, Fair 5%
Di Giacinto et al. (2021) [14]	2 years	Prospective randomized study	43 (12-14 years)	IMN	23	12.86 ± 11.77	15M 8F	Rt:8 Lt:15	FOOSH: 8, BI: 4, MVA: 4, Sports: 7	10.4 ± 1.22	No significant difference in the objective quality of life and wrist function
				Plating	20	13.02 ± 0.64	13M 7F	Rt:7 Lt:13	FOOSH: 5, RTA: 5, MVA: 5, Sports: 5	11.4 ± 1.34	

Discussion

Fractures of both the radius and ulna are common injuries sustained in the paediatric age group. An extensive test survey has shown that the bulk of these fractures can be managed non-operatively with cast immobilization, which has been very successful [6]. Injuries not suitable for conservative management were treated primarily by surgical intervention or were subsequently operated on following an unsuccessful trial of conservative management [7,17]. This operative intervention essentially involves either IMN or plating, both of which are acceptable surgical options in the paediatric age group. However, within the adult population, orthopaedic surgeons often opt for plating as the treatment of choice [17]. Despite both techniques being suitable for treating both-bone forearm fractures in the paediatric population, there still exist considerable controversies as to which is superior [2].

This review is a critical analysis of six cohort studies consisting of two prospective, three retrospective and a non-randomized trial, all with clearly stated aims. A total of 409 participants were involved from across the different studies. All with the exception of Barua et al. included older children aged 10 years and above who have limited remodelling capability [13]. An age range of 5-15 years in the Barua study is more representative of the paediatric population but does not explore a subgroup analysis of the younger cohorts who have the greatest remodelling potential [13]. Most of the included studies had a higher mean age with the plating group when compared with nailing. This is suggested to reflect a consideration to treat older children with less remodelling capabilities with plating in order to achieve a better anatomical reduction. The rationale behind the choice of procedure was not justified in any of the studies and the preferred choice was based on the preference of the operating surgeon. Appropriate qualitative methodology was employed in each of the studies with clear recruitment criteria. The study examined different treatment modalities including the hybrid technique which involves the use of plating for the radius and nailing for the ulna [12]. There was consistency in the inclusion and exclusion criteria and clear indications for surgery in all the studies.

Functional and Radiological Outcomes

The most recent noteworthy study was a systematic review by Patel et al. on functional outcomes and complications in both-bone diaphyseal forearm fractures in children [15]. It was a review of eight retrospective comparative studies of level III and IV evidence, which demonstrated similar functional outcomes, radiological outcomes and complications between IMN and plating. Patel and colleagues also identified a shortened operative time, better cosmetic outcomes and straightforward hardware removal with the intramedullary participants [15]. Similarly, a meta-analysis conducted on 13 studies which were randomized controlled trials and cohorts, involving both adults and children produced identical outcomes on function, radial bow magnitude and time to union [16]. It is also worth mentioning that the results are similar to the meta-analysis conducted by Zheng et al. in the adult population [1]. Findings are comparable with those in this review which has shown no statistically significant difference in the functional and radiological outcomes.

Half of the studies used an objective standardised scoring system created by Price et al. which compared the injured arm with the contralateral arm [9]. These studies showed a trend for higher rates of excellent and good functional outcomes which did not favour any of the techniques [1,12,13]. This differs from data obtained from Baldwin’s systematic review and meta-analysis of observational studies that favours an excellent functional outcome with the plating group but overall showed no statistical significance between both techniques [2]. The probability of having an excellent outcome was demonstrated by Flynn et al. as inherently more in younger children when they compared a subgroup of younger and older children that underwent nailing [7]. However, this was not replicated in plating participants in his study nor was it apparent in this review as patients were unevenly distributed favouring the older children [7]. One study used a clinician-completed scoring system to assess the level of disability in the wrist, assess pain, range of motion and grip strength. It also employed a reliable patient-reported outcome measure, Quick DASH, which is a validated scoring system [14]. Not all included studies evaluated functional outcomes nor had sufficient data on how they assessed this outcome. A systematic review conducted in 2014 by Baldwin and colleagues strengthened existing studies that upheld identical functional outcomes in a range of motion between nailing and plating in children with both-bone diaphyseal forearm fractures [2]. Moreover, a meta-analysis carried out by Zhao et al. amongst children and adults with related injuries analysed data from three studies and demonstrated similarities in loss of forearm rotation in both the nailing and the plating group (RR=1.27, P=0.508) [16].

Radiological evaluation was also considered using the time to union, maximum radial bow and location of the maximum radial bow. The radiological union had been expressed across all studies and was based on the formation of bridging callus on anteroposterior and lateral radiographs. There was no standardisation to the timing of follow-up X-rays in all studies. Compared to plating, the nailing group in this review revealed an increase in time to union in all but one study. Although significant, overall this difference is thought not to be clinically relevant and poses no statistical significance when both groups are weighed against each other. Similar outcomes were portrayed in a study which attributed findings to a bias in participant distribution involving older children > 10 years with an expressed hazard ratio of 1.89 for plating relative to nailing (95% CI, P=0.01) [4]. This was worth considering given that Flynn et al. had earlier argued that there exists a linear correlation between patient age and the time to union, with younger children showing a faster time to union [7].

Existing evidence also found a similar effect with plating and nailing groups in time to union as well as union rate. An analysis of five different studies by Zhao et al. showed that plating when compared with nailing did not significantly increase the union rate [RR 0.95, 95% CI (0.85, 1.05), (P = 0.312)] [16]. This was consistent in a subgroup analysis conducted in the same study for both children and adults [16]. Results are comparable to the findings of this systematic review which analysed an older sample population with a mean age > 10 years and concluded that there was no significant difference in time to union in both groups but acknowledged a shorter operative duration with the nailing technique. Other parameters used to evaluate radiological outcomes like radial bow location and magnitude were expressed in half the reviewed studies and were determined using the method described by Firl and Wunsch [18]. They reported normal values for children as 7.21% ± 1.03% and 60.39% ± 3.74% representing magnitude and bow location respectively. Analysis of the studies using this method concluded that plating provided a better restoration of anatomical radial bow compared to nailing. Schemitsch and Richards determined that restoration of the radial bow is of particular standing in re-establishing the normal forearm anatomy, thereby allowing for an improved grip function as well as better forearm rotation [19]. Some studies suggest that changes in either the magnitude or location of the radial bow would result in the loss of forearm rotation. A review of available data acknowledges that a better radial bow and magnitude was achieved using plating in comparison to nailing. However, it also concluded that there was no statistically significant difference in radial bow magnitude and location between the two groups [16].

Complications

Complications are a primary drawback that must be considered by patients undergoing surgery. They are largely categorised by most authors into major and minor complications. Major complications include non-union, delayed union, mal-union, and implant failure as mentioned in a number of studies [4,8,12,14]. On the other hand, this review refers to minor complications such as SSI, radial nerve palsy, as well as bursitis. Only one study employed the modified Clavien-Dindo classification scheme, a validated and rational scoring system which reports complications in order of increasing severity from one to five [4]. The summative risk of developing a complication when weighing both techniques was similar despite the different rates of various complications across the studies. There was no compelling difference in an attempt on calculating a risk ratio using the Mantel-Haenszel random effect model [RR 1.48, 95% CI (0.87, 2.52), (P = 0.15)]. This finding was attributed to the relatively small sample size of the plating participant when compared to nailing which had over 14% more patients. Data from Patel et al. has correlated a significant decrease in complication rate with the nailing group [15]. This differs from the meta-analysis conducted by Zhao et al. with similar complication outcomes in both nailing and plating for children, but lower complication rates for adults with the nailing group [16]. Similar to this review, Baldwin and colleagues conducted a meta-analysis that demonstrated no significant difference between both techniques (P = 0.68) but illustrated an effect size in favour of plating [2].

SSI is one of the widely reported complications. Some studies have associated it with the nailing group; its predominance within this group was traced to nail insertion at the point of entry as well as the prominence of metalwork which can cause skin irritation [3]. Other studies are of the opinion that a larger surgical site exposure and soft tissue irritation are responsible for infection in patients undergoing surgical plating [16]. Some plating participants have been noted to develop an infection following the removal of metalwork [20]. One participant in the retrospective study conducted by Flynn et al. recorded re-operation 6 months post-plating for implant removal secondary to infection [21].

This review also noted that delayed union and non-union are relatively more common than previously documented. The rationality behind delayed union is suggested by some authors to include opening the fracture site to encourage reduction, periosteal stripping and blood circulation disruption, especially with plating [3,7,12]. Other factors associated with delayed union include the use of a thick nail which can disrupt the fracture, as well as the retrograde nailing technique [3]. Baldwin et al. found that delayed union was common with the nailing group (5.5%) compared to plating (0.8%) [2]. However, they concluded that the difference was not statistically significant (P = 0.06) [2]. Outcomes of a multi-centre retrospective study conducted by Schmittenbecher et al. (2008) established that there was a high risk of delayed union with plating [22]. In contrast, more recent studies have demonstrated that delayed union is more common with IMN, although this has not been proven to be statistically significant [1].

For the purpose of this review, non-union was described based on radiological and time-related criteria as persistence of fracture greater than 6 months with no change in fracture callus for up to 3 months. Studies have reported non-union as a relatively uncommon complication within the paediatric population. Existing evidence has shown only a few percentages may develop this complication; 1.7% of participants treated with plating and 2.4% treated with IMN [2]. Non-union was hypothesised to develop following overzealous soft tissue dissection, periosteal stripping, distraction at fracture ends and a large medullary cavity of the radius in older children [12]. Although the numbers favour plating the differences has not been shown to bear statistical significance (P = 0.74) [2]. Similarly, this is demonstrated in three of six studies in this review with data distribution showing a higher risk with nailing patients compared with plating.

Mal-union is a rare complication following both plating and nailing. It has been reported with a higher incidence in conservatively managed diaphyseal forearm fractures [9]. Although other studies have noted the possibility of mal-alignment with operative intervention. These cases have been associated with nailing and there are worries that this may affect pronation and supination, especially with distal forearm fractures [15]. It is also worth noting that no residual deficit in performing activities of daily living was recorded by nailing patients who went on to have mal-union [16]. Only one case of mal-union has been documented in this review by Di Giacinto et al. at 8.7 ± 2 weeks during follow-up and after removal of Kirchner wire [14]. Other complications discussed include nerve palsy which had a prevalence amongst plating patients. Nerve palsy was also found to be associated with nailing following irritation at the entry site of the hardware [2]. Overall, most palsy were recorded to have resolved at 3 months follow-up visits for both groups; so, earlier differences in data held no clinical significance [1,15,16]. Re-fracture rates in the ipsilateral limb were similar between nailing and plating, with most cases occurring after early removal of metal work or following a repeat fall [1,12,14].

An important consideration while evaluating both techniques was the operating time. The mean surgical time from data retrieved in this study ranged from 32.6 to 54 minutes and 54.8 to 69.7 minutes for IMN and plating respectively. This was presumably a result of the different levels of expertise of the surgeons which were not uniform across the board. However, it was generally agreed by most authors that nailing significantly reduced the operative time [5,23,24]. Findings are consistent with this review and this is touted as a major advantage of this technique.

Limitations

This review has been pervaded by a number of flaws that should be considered when interpreting the results. A critique of this review showed 6 included studies all of which had a relatively small sample size and no power calculation to determine the probability of type II error. This can amplify the treatment effect when compared with larger sample studies. Furthermore, all the studies were level III retrospective or prospective studies which are highly subjective to cofounding. There was no mention of blinding or randomization in any of the studies. This increases the likelihood of selection bias which can probably be corrected by randomized studies. A number of the included studies did not consider the level of expertise of the operating surgeon, or the number of surgeons per study. Scoring systems were employed to evaluate functional and radiological outcomes. However, only one paper used a validated scoring scheme. Some studies had incomplete data relevant to the outcomes stated in the methodology, while others lacked either a standard deviation or mean and median scores. This heterogenicity encountered in the pattern of reported outcomes meant that data could not be extracted for a meta-analysis nor statistically accommodated as this could produce misleading results.

## Conclusions

This systematic review provides evidence that supports a similar functional and radiological outcome as well as complication rate in both nailing and plating in children with both-bone forearm fractures. Following this review, we concluded that both surgical options are effective treatment modalities and should be considered in managing both-bone forearm fractures in children. However, we acknowledge that finite data is insufficient to determine which surgical technique confers a better outcome. Thus, higher-level trials with larger sample sizes are recommended.
